# Supplementary material for: Comparative Transcriptome Investigation of Nosema ceranae Infecting Eastern Honey Bee Workers
Source: Insects. 2022 Feb 28;13(3):241. doi: 10.3390/insects13030241 (PMC8952433; doi:10.3390/insects13030241)
Supplement: Supplementary file 1 [file insects-13-00241-s001.zip › insects-1538265-supplementary.pdf]

Table S1. Summary of RNA-seq datasets (*Apis cerana*)

Sample	Raw reads	Clean reads	Q20 (%)
Ac7CK	174,700,032	171,868,061	94.96%
Ac7T	205,297,946	200,570,776	94.58%
Ac10CK	124,216,829	121,949,977	94.60%
Ac10T	99,030,788	97,432,267	94.91%

Table S2. Summary of RNA-seq datasets (*Nosema ceranae*)

Sample	Raw reads	Clean reads	Q30 (%)
NcCK	416, 156, 600	210, 824, 312	92.63%

Table S3. Primers used in this study

Primer name	Primer sequence (5'-3')	Product size (bp)	Purpose
Nc-F	CGAGCGGTTTCCCATCTCAGTA	76	PCR
Nc-R	AAAACACCGGAACTCGTCAGCT		
Na-F	CCATTGCCGGATAAGAGAGT	401	PCR
Na-R	CACGCATTGCTGCATCATTGAC		
XM_002994928.1-F	ATTTCAGTAATGCCAACA	200	qPCR
XM_002994928.1-R	CACTCCTCTATCTCCACC		
XM_002996303.1-F	CAGGTGTAATGGTTTT	136	qPCR
XM_002996303.1-R	CCTCTGTTTCTTGGTA		
XM_002996348.1-F	TAAAGGGGGAGATGCTGCT	165	qPCR
XM_002996348.1-R	TTTTCTGGTCCTCTTGGGG		
XM_002995446.1-F	CTTTCCACTTTATGTCT	249	qPCR
XM_002995446.1-R	CTTTCCACTTTATGTCT		
XM_002996294.1-F	CACTCACTTACAGCAACA	244	qPCR
XM_002996294.1-R	TTAGATAAACCAACCAAA		
XM_002996299.1-F	ACGACTCTTGTTCTTGCT	102	qPCR
XM_002996299.1-R	AATGTCTATGTCCCCCTC		
XM_002995682.1-F	ATTGCTAAGGTAGAAAGA	214	qPCR
XM_002995682.1-R	TAAAAGAATAAGAGAGTG		
XM_002996538.1-F	AGCAAAGGTTGAGAGAAA	168	qPCR
XM_002996538.1-R	ATAAATGAGAACGGAAGG		
XM_002996655.1-F	TACCTACGGAAGATGAG	143	qPCR
XM_002996655.1-R	TAAAAATGAATACACAA		
XM_002996468.1-F	AAAGGCAAGTAAAGACC	183	qPCR
XM_002996468.1-R	TTCAAAACAGTAAAATC		
XM_002996253.1-F	ATTACTGTTGTCTCCCA	209	qPCR
XM_002996253.1-R	AACTTACCACTTTCCCC		
